# Draft genomes of three closely related low light-adapted *Prochlorococcus*

**DOI:** 10.1186/s12863-022-01103-4

**Published:** 2023-02-24

**Authors:** Jessie W. Berta-Thompson, Elaina Thomas, Andrés Cubillos-Ruiz, Thomas Hackl, Jamie W. Becker, Allison Coe, Steven J. Biller, Paul M. Berube, Sallie W. Chisholm

**Affiliations:** 1 Department of Civil and Environmental Engineering, Massachusetts Institute of Technology, Cambridge, MA 02139 USA; 2 Department of Research and Conservation, Denver Botanic Gardens, Denver, CO 80206 USA; 3 School of Oceanography, University of Washington, Seattle, WA 98195 USA; 4 Groningen Institute of Evolutionary Life Sciences, University of Groningen, Groningen, 9700 CC The Netherlands; 5 Department of Science and Mathematics, Alvernia University, Reading, PA 19607 USA; 6 Department of Biological Sciences, Wellesley College, Wellesley, MA 02481 USA; 7 Department of Biology, Massachusetts Institute of Technology, Cambridge, MA 02139 USA

**Keywords:** *Prochlorococcus*, Microdiversity, Enrichment culture, Genome, Marine microbiology, North Pacific

## Abstract

**Objectives:**

The marine cyanobacterium *Prochlorococcus* is a critical part of warm ocean ecosystems and a model for studying microbial evolution and ecology. To expand the representation of this organism’s vast wild diversity in sequence collections, we performed a set of isolation efforts targeting low light-adapted *Prochlorococcus*. Three genomes resulting from this larger body of work are described here.

**Data description:**

We present draft-quality *Prochlorococcus* genomes from enrichment cultures P1344, P1361, and P1363, sampled in the North Pacific. The genomes were built from Illumina paired reads assembled de novo. Supporting datasets of raw reads, assessments, and sequences from co-enriched heterotrophic marine bacteria are also provided. These three genomes represent members of the low light-adapted LLIV *Prochlorococcus* clade that are closely related, with 99.9% average nucleotide identity between pairs, yet vary in gene content. Expanding the powerful toolkit of *Prochlorococcus* genomes, these sequences provide an opportunity to study fine-scale variation and microevolutionary processes.

## Objective

Inhabiting the oligotrophic open oceans, the cyanobacterium *Prochlorococcus* is the most abundant photoautotroph on Earth, influencing local trophic flow and global biogeochemical cycling [1, 2]. A minimalist adapted to its low-nutrient environment [3, 4], *Prochlorococcus* is unique among cyanobacteria for its small cells, 0.5 – 1.0 µm diameter [5, 6], and small genomes, 1.6 – 2.7 Mb [7]. As a model system, *Prochlorococcus* has revealed ecological structure and adaptation at many scales (e.g., [7–12]).

*Prochlorococcus* diversity is organized into phylogenetic clades, sorted into low light-adapted (LL) and high light-adapted (HL) groups based on ecological, genomic, and physiological patterns [7, 12, 13]. While HL clades are more abundant [9, 14], LL *Prochlorococcus* include lineages adapted to uniquely challenging conditions, including anoxic zones [15], seasonal mixing [14], and light limitation in the deep euphotic zone [12]. LL strains have larger genomes with substantial flexible genome content [7]. Known wild diversity among LL *Prochlorococcus* far exceeds culture collections [7], and culturing this organism can be unpredictable [16].

In this context, we performed isolation and sequencing efforts targeting LL *Prochlorococcus* during a North Pacific sampling opportunity. This work resulted in previously described strains [17–19] and the three LLIV clade genomes described here: P1344, P1361, and P1363. To date, these new genomes have been used in studies on novel mobile genetic elements [20] and diversity in *Prochlorococcus* enrichment cultures [19]. These genomes represent a set of sympatric, closely related strains, supporting the study of microevolution in the growing *Prochlorococcus* sequence collection.

## Data description

We present draft *Prochlorococcus* genomes from enrichment cultures P1344, P1361, and P1363 (Dataset 1) [21–23]. They come from a single sample collected in the North Pacific from 150 m at Station ALOHA (22.75°N, 158°W), June 2013 [24, 25]. Isolation protocols [16] were tuned to enrich for LL *Prochlorococcus*. Data file 1 [26] provides detailed methods; Table 1 lists datasets. Previously described strains from the same project [17–19] are described in Data file 2 [26]. After 1.5 years of subculturing, enrichments P1344, P1361, and P1363 each stabilized to a single internal transcribed spacer rRNA (ITS) sequence [27], an indicator of unialgal *Prochlorococcus* cultures [16, 28]. Because the time from sea to genome was shorter than for previously sequenced enrichment cultures (e.g., 5–20 years [28]), we followed a naming convention for *Prochlorococcus* enrichments [29]. The three ITS sequences, in the LLIV clade, were identical to each other, strains MIT1312 and MIT1327 (additional co-isolates from the same sample [17]), and MIT1227 (from Station ALOHA one year earlier [30]).

**Table 1:** Overview of data files/data sets

Label	Name of data file/data set	File types (file extension)	Data repository and identifier (DOI or accession number)
Dataset 1a	Draft genome for *Prochlorococcus* P1344	Fasta sequence files (.fsa) and Genbank flatfile annotations (.gbff)	NCBI Genbank: JABBYR000000000.1 https://identifiers.org/nucleotide:JABBYR000000000.1 [21]
Dataset 1b	Draft genome for *Prochlorococcus* P1361	Fasta sequence files (.fsa) and Genbank flatfile annotations (.gbff)	NCBI Genbank: JABBYP000000000.1 https://identifiers.org/nucleotide:JABBYP000000000.1 [22]
Dataset 1c	Draft genome for *Prochlorococcus* P1363	Fasta sequence files (.fsa) and Genbank flatfile annotations (.gbff)	NCBI Genbank: JABBYQ000000000.1 https://identifiers.org/nucleotide:JABBYQ000000000.1 [23]
Dataset 2a	Raw sequencing reads for P1344 enrichment	Fastq sequence file (.fastq)	NCBI Sequence Read Archive: SRR11497176 https://identifiers.org/insdc.sra:SRR11497176 [31]
Dataset 2b	Raw sequencing reads for P1361 enrichment	Fastq sequence file (.fastq)	NCBI Sequence Read Archive: SRR11497178 https://identifiers.org/insdc.sra:SRR11497178 [32]
Dataset 2c	Raw sequencing reads for P1363 enrichment	Fastq sequence file (.fastq)	NCBI Sequence Read Archive: SRR11497177 https://identifiers.org/insdc.sra:SRR11497177 [33]
Dataset 3a	Prokka genome annotations for *Prochlorococcus* P1344	Genbank flatfile annotations (.gbf)	figshare: https://doi.org/10.6084/m9.figshare.12675410 [26]
Dataset 3b	Prokka genome annotations for *Prochlorococcus* P1361	Genbank flatfile annotations (.gbf)	figshare: https://doi.org/10.6084/m9.figshare.12675410 [26]
Dataset 3c	Prokka genome annotations for *Prochlorococcus* P1363	Genbank flatfile annotations (.gbf)	figshare: https://doi.org/10.6084/m9.figshare.12675410 [26]
Dataset 3d	Prokka genome annotations for *Prochlorococcus* MIT1227	Genbank flatfile annotations (.gbf)	figshare: https://doi.org/10.6084/m9.figshare.12675410 [26]
Dataset 3e	Prokka genome annotations for *Prochlorococcus* MIT1312	Genbank flatfile annotations (.gbf)	figshare: https://doi.org/10.6084/m9.figshare.12675410 [26]
Dataset 3f	Prokka genome annotations for *Prochlorococcus* MIT1327	Genbank flatfile annotations (.gbf)	figshare: https://doi.org/10.6084/m9.figshare.12675410 [26]
Dataset 4a	Enrichment assembly for P1344	Fasta sequence file (.fasta)	figshare: https://doi.org/10.6084/m9.figshare.12675410 [26]
Dataset 4b	Enrichment assembly for P1361	Fasta sequence file (.fasta)	figshare: https://doi.org/10.6084/m9.figshare.12675410 [26]
Dataset 4c	Enrichment assembly for P1363	Fasta sequence file (.fasta)	figshare: https://doi.org/10.6084/m9.figshare.12675410 [26]
Data file 1	Detailed methods	Document (.pdf)	figshare: https://doi.org/10.6084/m9.figshare.12675410 [26]
Data file 2	Genome summary information	Microsoft Excel file (.xlsx)	figshare: https://doi.org/10.6084/m9.figshare.12675410 [26]
Data file 3	Enrichment contig characteristics	Microsoft Excel file (.xlsx)	figshare: https://doi.org/10.6084/m9.figshare.12675410 [26]
Data file 4	Genome contig characteristics	Microsoft Excel file (.xlsx)	figshare: https://doi.org/10.6084/m9.figshare.12675410 [26]
Data file 5	Annotation information	Microsoft Excel file (.xlsx)	figshare: https://doi.org/10.6084/m9.figshare.12675410 [26]
Data file 6	Overview of other organisms in enrichment BLAST results	Microsoft Excel file (.xlsx)	figshare: https://doi.org/10.6084/m9.figshare.12675410 [26]
Data file 7	Genome and enrichment bin completeness and taxonomy	Microsoft Excel file (.xlsx)	figshare: https://doi.org/10.6084/m9.figshare.12675410 [26]
Data file 8	Protein ortholog clusters for identical ITS group	Microsoft Excel file (.xlsx)	figshare: https://doi.org/10.6084/m9.figshare.12675410 [26]
Data file 9	Genome comparisons for identical ITS group	Microsoft Excel file (.xlsx)	figshare: https://doi.org/10.6084/m9.figshare.12675410 [26]

Genomic libraries were prepared as in [34] from bulk enrichment DNA and sequenced with Illumina MiSeq V3 at the MIT BioMicroCenter [35] with 300 base paired reads. Raw reads are available in the NCBI Sequencing Read Archive (Dataset 2) [31–33]. Quality-trimmed reads were assembled de novo with SPAdes v.3.1.1 [36]. Enrichment contigs were screened with blastn [37] against the NCBI nt database to separate out *Prochlorococcus* sequences (Data file 3) [26]. Contigs with at least 500 bases, top BLAST hits to *Prochlorococcus* (Data file 4) [26], and at least 2 × kmer coverage were selected to produce the genomes. For P1344, P1361, and P1363, respectively, genomes consist of 106, 45, and 66 contigs, with average read coverage depths 82x, 57x, and 67x [38] and genome sizes 2.47 Mb, 2.51 Mb, and 2.56 Mb, similar to other LLIV genomes (Data file 2) [26].

For initial assessments, we annotated the genomes with Prokka [39] (Dataset 3, Data file 5) [26]. Genbank annotations come from the NCBI automated annotation pipeline (Dataset 1) [22–24]. Enrichment assembly (Dataset 4 [26]) BLAST and metagenomic binning results (Data files 1, 3, 6, 7) [26] show the presence of copiotrophic marine bacteria at lower coverage than *Prochlorococcus*, with partially recovered genomes [40–43]. These include *Alteromonas* and *Marinobacter*, groups previously studied in co-culture with *Prochlorococcus* that can enhance its growth or survival in culture [18, 19, 44–46]. Comparisons among P1344, P1361, P1363, and the three other genomes with the same ITS (Data files 1, 8, 9) [26] support the idea that they represent similar but distinct strains, with average nucleotide identity from 99.9% ± 0.7% to 100.0% ± 0.1% s.d. [47], 103 – 3,854 SNPs in pairwise alignments [48, 49], and 32 – 132 distinct genes in pairwise ortholog group comparisons [50]. While mostly without predicted functions, these variable genes include a pilus-related protein and a member of the cytochrome c family, located near contig ends (Data files 1, 5) [26]. This scale of variation will support the study of recent *Prochlorococcus* evolution.

## Limitations

The enrichment cultures described here were lost during maintenance and are no longer available. These genomes came from enrichments rather than clones, representing a snapshot in time of a likely single dominant strain in each culture and an associated heterotrophic community (Data file 1) [26]. That these genomes are not linked to existing cultures and did not come from clonal isolates limits downstream use and requires caution in interpretation, but not more so than sequences derived from single cell methods or metagenomics, and with the benefit of more complete genomes. With care, these new genomes still have the potential to contribute useful insights on the nature and mechanisms of fine-scale evolution in *Prochlorococcus*.

## Data Availability

The genomic data described in this Data note can be freely and openly accessed at the NCBI Genbank database under Bioproject PRJNA623403, with accession numbers JABBYR000000000.1 (P1344), JABBYP000000000.1 (P1361), and JABBYQ000000000.1 (P1363) [21–23]. Raw enrichment sequencing reads are available in the NCBI Sequencing Read Archive under accession numbers SRR11497176 (P1344), SRR11497178 (P1361), and SRR11497177 (P1363) [31–33]. Detailed methods and supportive datasets are available on figshare at https://doi.org/10.6084/m9.figshare.12675410 [26].

## Abbreviations

HL: High light-adapted
LL: Low light-adapted
ITS: Internal transcribed spacer between the 16S and 23S ribosomal RNA genes
NCBI: National Center for Biotechnology Information
